# Multiplexed lighting system using time-division multiplexing

**DOI:** 10.1007/s12652-022-03778-0

**Published:** 2022-03-11

**Authors:** Yuki Ban, Koichi Ota, Rui Fukui, Shin’ichi Warisawa

**Affiliations:** Graduate School of Frontier Sciences, The University of Tokyo, 5-1-5 Kashiwanoha, Kashiwa, Chiba 2778563 Japan

**Keywords:** Intelligent lighting system, Multiplexed lightning environment, Indoor environment improvement, Controllability of lighting

## Abstract

Improvements in lighting and other indoor environmental conditions have gained considerable attention in different areas, including health and economics. Controlling the lighting environment is essential because, among the indoor factors, visual stimulation affects numerous human characteristics. Further, visual stimulation, including peripheral vision, affects people differently. Therefore, to improve the indoor environment with multiple occupants, each occupant must have an independent lighting environment. However, this cannot be achieved through conventional approaches. In this study, we propose a multiplexed lighting environment that can simultaneously realize multiple mutually independent lighting environments within a single space. We developed the proposed system using time-division multiplexing and conducted an experiment to clarify the influence of light multiplexing on human behavior and impression of the indoor environment. The experimental results showed that the proposed method changed the lighting operations of the users and improved their impression of the lighting environment. Furthermore, the proposed method provides a desirable lighting environment for all people within a single space, even when people in the same space desire different lighting environments.

## Introduction

Improvements in lighting and other related aspects of indoor environments have attracted considerable attention from researchers in different fields, such as health and economics (Al Horr et al. 2016). Studies on improving the desirability of a space (e.g., an office) by improving its indoor environment have been actively conducted in recent years. Many studies have highlighted that factors such as the degree of concentration, productivity, and fatigue are affected by visual stimuli (van Duijnhoven et al. 2019). In addition to the visual stimuli in our central vision, which we consciously recognize, the stimuli available for peripheral vision, which we process unconsciously, also affect these factors (Houser et al. 2002; Parpairi et al. 2002). Therefore, several research groups have attempted to design lighting environments that can help control the visual stimuli surrounding an occupant, including the peripheral vision, and improve the desirability of an indoor space.

Some studies have suggested that the influence of visual stimuli varies from person to person (Juslén et al. 2005). To improve the desirability of all occupants in a space, it is necessary to present a different lighting environment for each individual. However, in a space with multiple occupants, the lighting environment for each occupant overlaps spatially. To resolve this overlap, it is necessary to realize multiple lighting environments in a single space, that is, a multiplexed lighting environment, to improve an indoor environment.

**Fig. 1:**
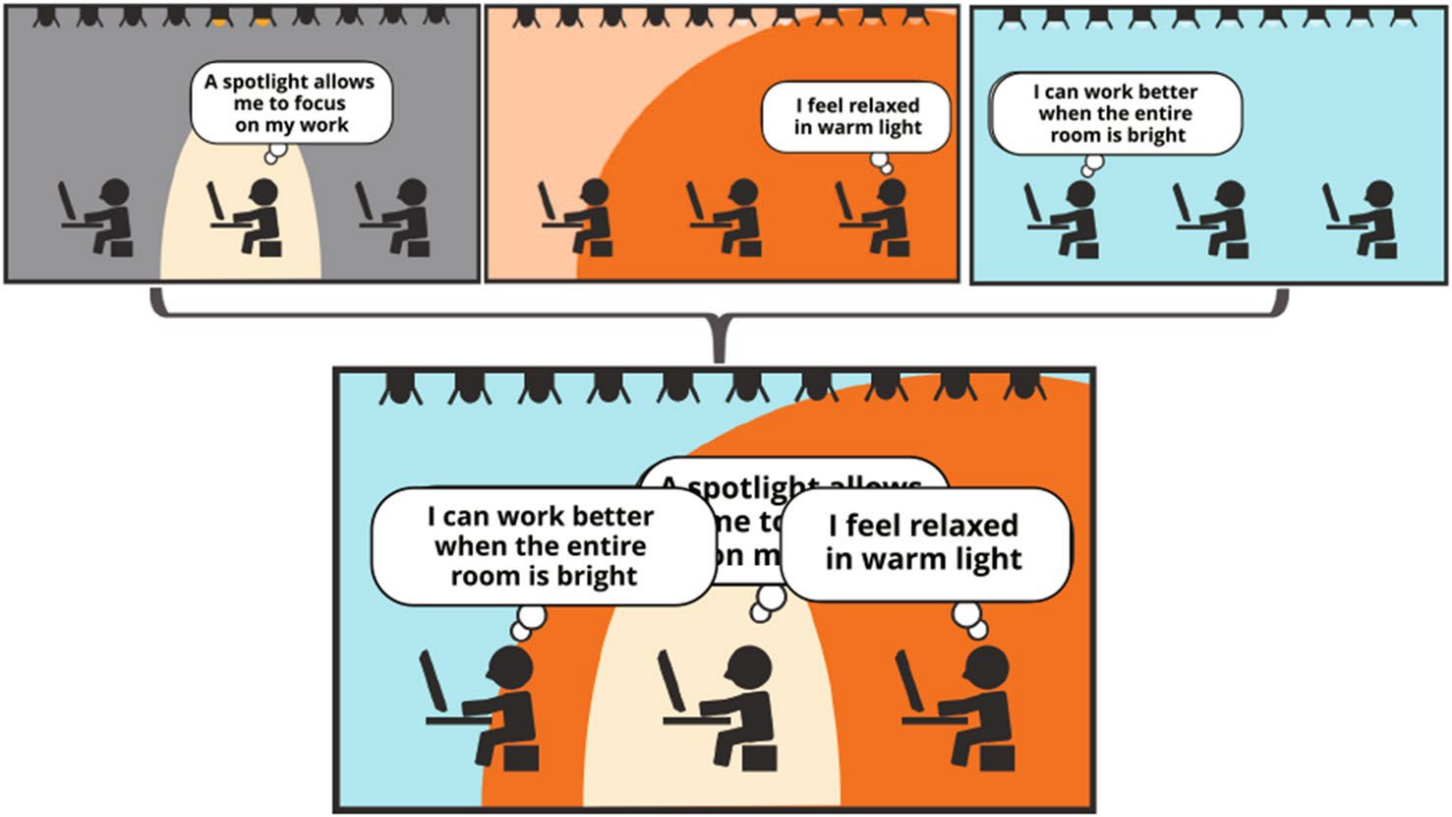
Conflicts occur when everyone attempts to satisfy the lighting environment in a single space

Several researchers have developed methods to present a more desirable lighting environment for each occupant in a single space. Systems that comprehensively control multiple lighting fixtures using computers are known as intelligent lighting systems. Using an intelligent lighting system, several researchers have developed methods for presenting desirable lighting conditions on the desktop of individual occupants  (Kaku et al. 2010; Tanaka et al. 2009; Shin and Woo 2009; Wang and Tan 2013; Bao et al. 2015). However, these methods cannot simultaneously provide multiple lighting environments in a single space. With these methods, even if we attempt to improve the satisfaction of all occupants within the entire space, the light environments in the peripheral field of view of the occupants will interfere with each other.

Therefore, in this study, we propose an approach for independently controlling the entire lighting environment within a single space, including peripheral stimulation for each occupant, and improving the satisfaction of all occupants. We call such independently controllable lighting a multiplexed lighting environment (Fig. 1). The multiplexed lighting environment simultaneously provides different lighting environments (intensity, color, and distribution of lighting, among other factors) for each occupant without spatial overlaps in a single space. For example, some occupants in a room may want to brighten their surroundings to immerse themselves in their work. Others may want to illuminate their surroundings with warm light for relaxation or to illuminate the entire room brightly to increase productivity.

To satisfy these varying states of illumination that are in conflict with each other, it is necessary to realize a system that allows people to experience different states concurrently in the same area. To accomplish this, we apply light multiplexing using a time-division scheme, which allows the transmission of multiple lights within a single space. This method utilizes the property by which human vision recognizes high-speed flashing lights as a single continuous light. and presents multiple rapidly blinking lights through the synchronized control of a shuttering glass-type device and light source. If this system can be realized, it is expected that a space can be more efficiently used by multiplexing lighting in an office or home environment. Using this system, individuals can adjust the lighting, including their surroundings, without worrying about the effect on others. Hence, this system has the potential to increase the satisfaction of the occupants. In addition, it can be applied to a wide range of areas that require lighting, including stage lighting.

We report a user study investigating the effect of this system on the occupants. To clarify the influence of our system on the behavior and mental state of an individual occupant, participants worked in an environment where they could control multiplexed lighting using our system, and we measured their behavioral and psychological indices.

This study was based on an abstract presented at SIGGRAPH Asia 2019 Emerging Technologies (Ota et al. 2019). In this paper, we organize the requirements and implementation of the proposed approach and describe a user study conducted to evaluate the effect of the system on occupants. The contributions of this study are as follows:

- We propose a “multiplexed lighting system” that realizes multiple lighting environments in a single space to simultaneously meet the different lighting environment demands of different occupants.
- We confirm that the “multiplexed lighting system” can be realized using light multiplexing through time division with an active shutter system.
- The user study reveals that the proposed system can eliminate the suppression of the desired lighting operation caused by sharing a lighting environment with others. Our system allows individuals in a single space to easily set their own desired lighting environment and improves their impression of the lighting environment.

## Related works

### Preferred illumination conditions

Light sources with different color temperatures, light intensities, and light spectra affect human circadian rhythms and physiological processes such as pupil diameter and melatonin production (Schratz et al. 2013; Lu et al. 2016). As described in the Introduction, the lighting environment affects various factors such as fatigue, concentration, emotion, and arousal. Veitch et al. created a model of psychological processes using mediated regression analysis. The authors concluded that environmental evaluations influenced by lighting conditions lead to environmental desirability, emotions, and health states (Veitch et al. 2008). Sugimoto reported that sympathetic nervous tension, or physical burden, manifested in heart rate and respiration is lowest at 320 lx and the psychological favorability is the highest at 1000 lx (Sugimoto 1981). Lighting environment factors, including brightness, distribution, and color temperature, affect humans. The preferred lighting environment depends on the situation and context, such as the preference for a high color temperature for work and a low color temperature for relaxation (Wang et al. 2017). Illumination at the center of the field of view (such as at a desktop), is not the only factor that affects occupants. Some studies have reported that the lighting of the entire room within the peripheral vision, such as the ratio of the brightness of the desktop to the ceiling and walls, can affect the impression of an indoor environment, including the brightness and spaciousness of the space (Houser et al. 2002), visual fatigue (Wang et al. 2015), and attention (de Vries et al. 2018).

Furthermore, it has been suggested that the effects of lighting environment vary from person to person (Boyce et al. 2000, 2006; Maierova et al. 2013). Juslen et al. stated that individuals have different preferred brightness rhythms, even within a particular week (Juslén et al. 2005). Other studies have suggested that simply allowing the occupant to operate the lighting can improve the assessment of the indoor environment. Occupants prefer an indoor environment where they can control lighting by themselves over an environment without such controllability (Juslén et al. 2005; Moore et al. 2004; Maniccia et al. 1999). Boyce et al. clarified that participants experienced a low level of difficulty in executing a task when they could adjust their lighting environment (Boyce et al. 2000). Afshari and Mishra reported that employees’ loss of control and self-determination over light can have negative psychological effects (Afshari and Mishra 2015). These studies evaluated the effects of lighting and lighting devices in scenarios where only a single person is in a space, such as a chamber. When multiple people are present in a room, it is unclear whether the controllability of the occupants has the same effect.

### Improvement of indoor lighting environment

An intelligent lighting system has been studied as an approach for presenting lighting to each person in an indoor environment with multiple occupants. A basic intelligent lighting system presents the intended illuminance in multiple specific areas by sensing the illumination and controlling the corresponding group of lights (Kaku et al. 2010; Wang and Tan 2013; Petrushevski et al. 2013). Research has been conducted to incorporate blinds and lighting into the control models. A lighting environment that considered energy savings and light comfort was designed (Xiong et al. 2019; Kandasamy et al. 2018). Although such a system can realize spatially unique illumination control, it cannot simultaneously satisfy two or more different demands for illuminance in a single area. We can control the illumination at the workspace using only spotlights and desk lights, and it is impossible for each person to experience different lighting conditions in all areas, including their peripheral vision. In addition, changing the lighting conditions at a certain point can change the lighting conditions in the surrounding area, as experienced by others.

The lighting preferences vary among individuals. Some studies have proposed a method for modeling users’ lighting preference profiles based on their control behaviors regarding characteristics such as activeness, dominance, lighting tolerance, and preference information (Despenic et al. 2017; Sadeghi et al. 2018). From this reason, some researchers have attempted to simultaneously satisfy the lighting requirements of multiple occupants within a space as accurately as possible. To enhance the satisfaction of all occupants within a space, some studies have devised methods for deriving a suitable lighting environment based on worker conditions, attributes, satisfaction, and behaviors (Shin and Woo 2009; Tanaka et al. 2009; Bando et al. 2018). These approaches estimate how severely the lighting environment affects each occupant and prioritize certain lighting conditions to improve the total satisfaction within a space. Another approach attempted to allow users to adjust both the task light and background light. The task light illuminates the personal spaces, while the background light is ambient. Kar et al. recommended lighting conditions based on personal preferences for the task light and collaborative preferences for the background light (Kar et al. 2019). However, as a result of prioritizing improvements in terms of overall satisfaction, the preferences of some occupants regarding the lighting environment might be discarded or ignored.

Another intelligent lighting approach controls a wider area of lighting conditions for each person by changing the seating arrangement and controlling lighting equipment (Bao et al. 2015). However, in such a system, it is difficult to respond to the demand-based control in the current scenario, because the seating arrangement needs to be changed whenever the lighting condition is changed. However, this approach is difficult to use when occupants in a room have completely different preferences in ambient lighting environments.

Hence, in this study, we developed a method allowing multiply independent control of the lighting environment in all areas of a space, thereby improving the satisfaction of all occupants simultaneously.

## Lighting environment multiplexing method

### Concept and hypotheses

Based on the challenges in the aforementioned studies, we propose an approach for independently controlling the entire lighting environment in a space, including the peripheral stimulation of each occupant, and for improving the satisfaction of all the occupants. Herein, we call such an independently controllable lighting environment a “multiplexed lighting environment” (Fig. 1).

We hypothesize that a multiplexed lighting environment in which users can control the lighting environment without affecting other occupants in a shared space would have the following effects on users. Boyce et al. (2000) and Hedge et al. (1995) found that an environment where occupants can control lighting conditions is preferred, and occupants select a lower lighting intensity than recommended for such an environment. In addition, Sakaue et al. showed that local lighting conditions in which the surroundings are dark in relation to the brightness of the work surface enhance the degree of concentration (Sakaue et al. 1997). It has also been reported that different types of work, such as simple work, creative work, and analytical work, have different preferred/appropriate combinations of color temperature and illumination that can improve their productivity and creativity (Lan et al. 2021; Ishii et al. 2018). Thus, the multiplexed lighting system may help change the lighting behavior of users and increase the number of times they adjust the lighting. Furthermore, a user may adjust the lighting conditions to be more suitable for concentrating. Based on the findings of previous studies (Veitch et al. 2008; Juslén et al. 2005; Afshari and Mishra 2015), we hypothesized that allowing users to manipulate room lighting independently would enhance their appreciation of the indoor environment and influence their emotions. The hypotheses stated in this section are summarized as follows:

- H1: The lighting operation of the user will change. Specifically, the number of lighting changes of a user will increase, and the lighting conditions around them will be darkened.
- H2: The appreciation of the indoor environment will increase, and users’ psychological conditions will be improved by allowing them to freely change their lighting environment.

In contrast to previous studies that attempted to increase the overall satisfaction of a space by zoning or scheduling ambient lighting to absorb some of the individual differences in lighting preferences (Bando et al. 2018; Kar et al. 2019; Trabelsi et al. 2016), this study provides each person with a completely independent lighting environment in the same space. Therefore, even in a relatively small area where ambient light zoning is difficult, it is possible to provide the desired lighting environment to all occupants without causing conflicts because of the differences in their lighting preferences and the appropriate lighting for different types of work. Furthermore, our proposed method can achieve this, even if the desired lighting environment differs completely among individuals. To verify these hypotheses, we constructed a prototype of the multiplexed lighting system and conducted a user study.

### Requirements

To construct a prototype system, we arranged the required functions and constraints. To independently control the lighting environment of the entire area of a space with multiple people, the following two functions need to be considered.

- The lighting should be controlled such that the illumination in any area of the space is experienced differently for each occupant.
- The illumination of a certain area changing as experienced by a particular occupant must not change the illumination of all other areas experienced by other occupants.

To apply the method to an actual indoor environment, it is necessary to satisfy the following constraints:

- It must not contain any elements that interrupt human indoor activities.
- The procedure used for changing the lighting conditions should not include actions that burden indoor activities, such as changing the seating arrangements.
- The application of the method must not change visual stimulation other than the lighting conditions.

### Approach

**Fig. 2:**
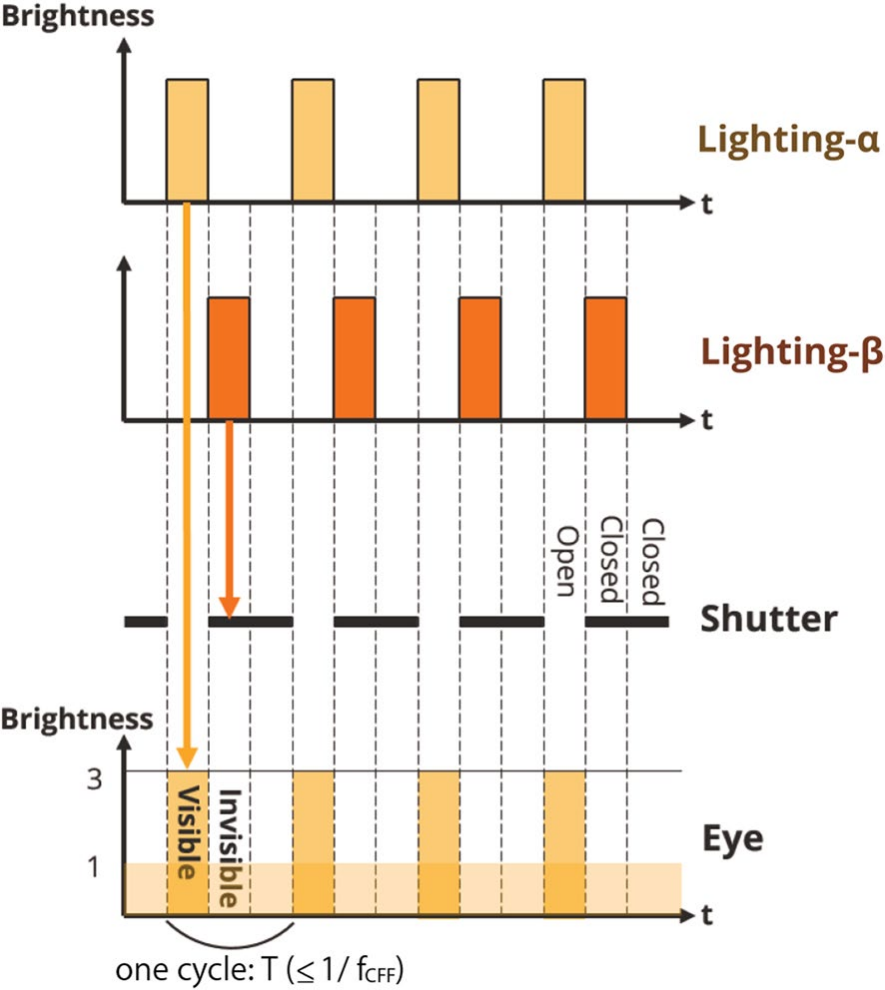
Principle of active shutter system (for a multiplicity of 3)

As mentioned in Sect. 2, existing intelligent lighting systems are unable to meet these requirements. Thus, we applied light multiplexing technology to realize multiple lighting environments where multiple mutually independent lighting states coexist in a single spatial area. In this paper, the number of independent light sources in a single spatial region is referred to as “multiplicity.” There are three main methods for multiplexing light in the visible-light region: time division, wavelength division, and polarization division. Approaches for multiplexing images using these techniques are widely known and used in projection mapping, virtual reality, and other areas (Woods 2012).

In this study, we propose a method for multiplexing the lighting environment using time-division multiplexing with an active shutter system. Compared with the other two multiplexing methods, our method does not have any color constraints, and the multiplicity of the lights can be increased depending on the control. Our system combines a rapidly flashing light source and shutters that repeatedly open and close at high speed, thereby realizing a multiplexed light from a single light source. Human vision has the property of recognizing rapidly blinking light as one continuous light. The minimum frequency at which flicker cannot be recognized is called the critical fusion frequency (CFF). The maximum CFF is 60 Hz, although it varies depending on the situation or individual (Sakurada et al. 2015). A blinking light beyond this frequency is recognized as light with an average brightness. Our method utilizes this property of human vision. Our system uses a rapidly flashing light source and the opening and closing of shutters at a speed synchronized with this flashing. In the case of Fig. 2, the light from lighting-$\alpha$ reaches the eye because lighting-$\alpha$ emits light while the shutter is open. However, the light from lighting-$\beta$ does not reach the eye because it emits light when the shutter is closed. Multiple shutters that are controlled to open only during mutually independent periods can provide independent lighting conditions.

### Implementation

**Fig. 3:**
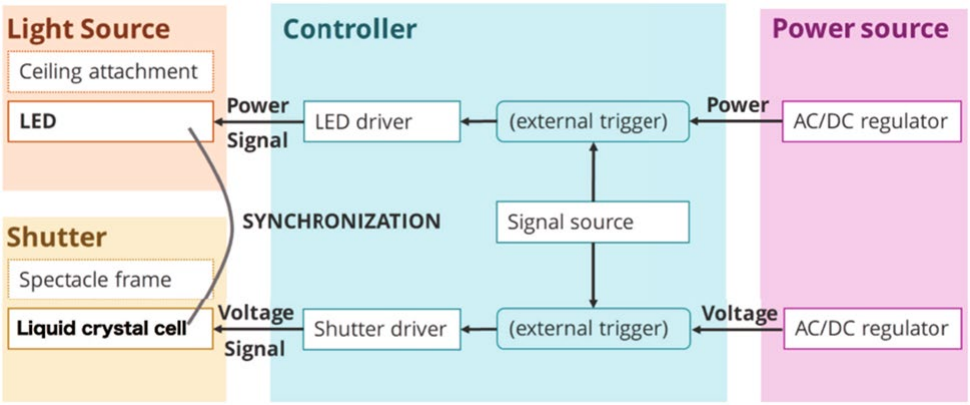
Diagram of the proposed system

Figure 3 shows the system diagram of our system. The speed at which the light blinks, that is, the speed at which we need to control the light source and shutter synchronously, is determined by the CFF and multiplicity. The multiplicity represents the expected number of occupants within the room. One cycle of control determined to satisfy the CFF is divided into several sections based on the multiplicity. Assuming that the CFF is 60 Hz and the multiplicity is *N*, the light source and shutter must be synchronously controlled at 60 *N* Hz or higher. We selected the materials for each part according to the following.

- Light source: Among the incandescent light bulbs, fluorescent lights, and LEDs, which are generally used for indoor lighting equipment, we adopted LEDs because they have the shortest time from power-on to lighting.
- Shutter: To block light, we used liquid crystal (LC) shutters instead of mechanical shutters. The opening and closing times of an LC shutter are several microseconds, which is applicable to our method, whereas the speed of a mechanical shutter is limited to several tens of times per second.
- Power source: The LED light source is turned on by applying an appropriate constant current. In addition, a liquid crystal shutter changes its transmittance by loading and releasing a voltage and then changes the light-shielding state. In this system, a constant-current power supply was used to control the lighting of the LED and a constant-voltage power supply was used to control the opening and closing of the liquid crystal shutter.
- Control signal source: Synchronous control can be programmed by inputting an ON/OFF control signal generated by the same microcontroller to the triggers that control the light source and shutter. An Arduino Mega 2560 R3 microcontroller board equipped with an ATMega 2560R3 was used.

**Fig. 4:**
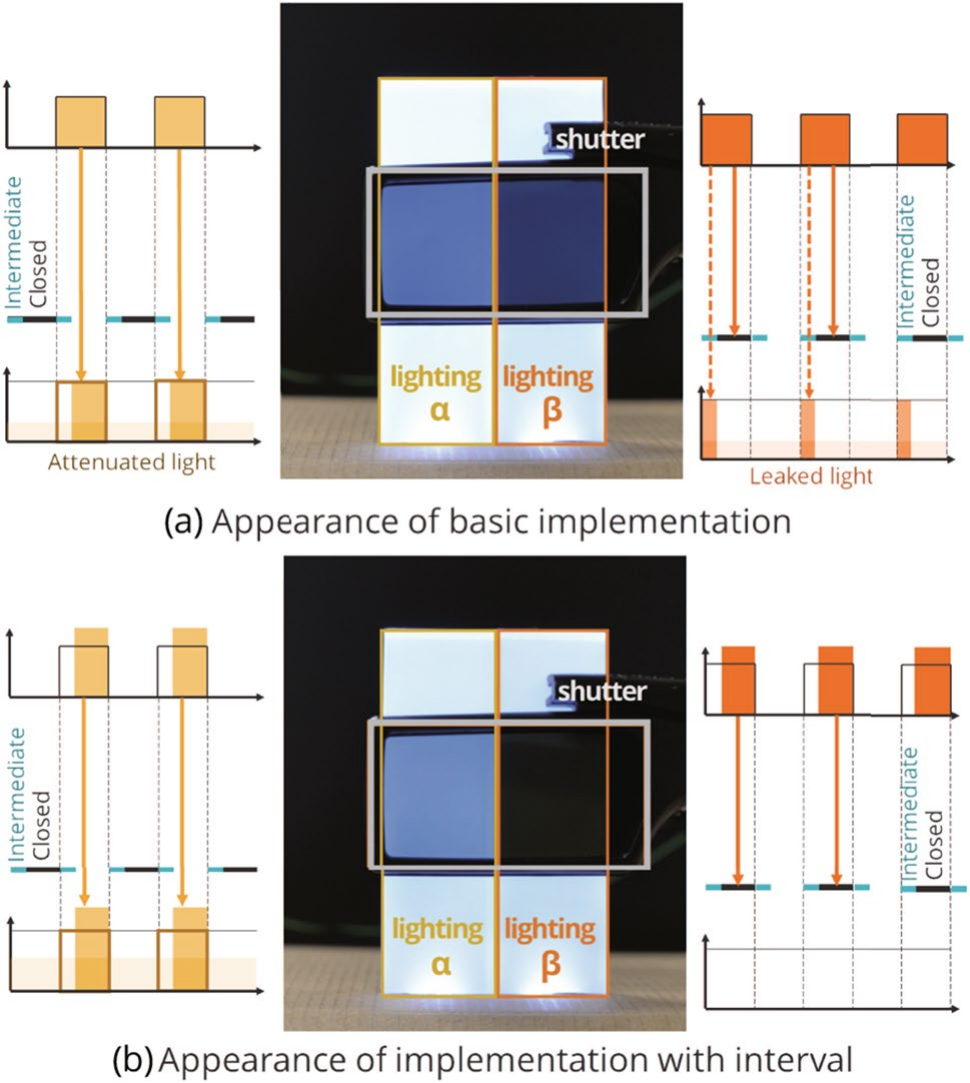
How light appears through the active shutter **a** without and **b** with interval periods

We conducted a preliminary verification of the lighting conditions using the implemented system. It took 0.4 ms for the shutter to change from completely closed to completely open and 1.0 ms from completely open to completely closed. To remove this effect, an interval period (1.0 ms) was provided for synchronous control of the shutter and light source. We verified the lighting multiplexing performance of the improved system (Fig. 4). It was shown that the system could pass the desired light without attenuation, and it could completely block out specified light (Ota et al. 2019).

The length of the interval period $\tau _{\mathrm{int}}$ should be set greater than either the time it takes for the shutter to completely open $\tau _{\mathrm{open}}$ or the time it takes to close completely $\tau _{\mathrm{close}}$ (Eq. 1).

1 $$\begin{aligned} \tau _{\mathrm{int}} > \max \{\tau _{\mathrm{open}}, \tau _{\mathrm{close}}\} \end{aligned}$$

We denote the multiplicity as *N*, the CFF as $f_{\mathrm{CFF}}$, and one cycle duration of control in the system as *T*. To avoid the perception of light flicker, it is necessary to set the duration of one cycle *T* shorter than the duration determined by the CFF; therefore, Eq. (2) is derived. For the length of interval period $\tau _{\mathrm{int}}$, the relationship in Eq. (3) holds.

2 $$\begin{aligned} T \le \frac{1}{f_{\mathrm{CFF}}} \end{aligned}$$

3 $$\begin{aligned} \tau _{\mathrm{int}} \ll \frac{T}{N} \le \frac{1}{N \cdot f_{\mathrm{CFF}}} \end{aligned}$$

Here, inequality $\ll$ indicates that the interval period should be less than a few percentage points of *T*/*N* to prevent the lighting period of the light source from being extremely short. That is, the multiplicity *N* is constrained by the time it takes to open and close the shutter as follows:

4 $$\begin{aligned} N \ll \frac{1}{f_{\mathrm{CFF}} \cdot \max \{\tau _{\mathrm{open}}, \tau _{\mathrm{close}}\}} \end{aligned}$$

The system developed and used in this study achieves a multiplicity of $N = 3$ using an LC shutter, as described in this section. This was sufficient to test hypotheses H1 and H2 in this study.

**Fig. 5:**
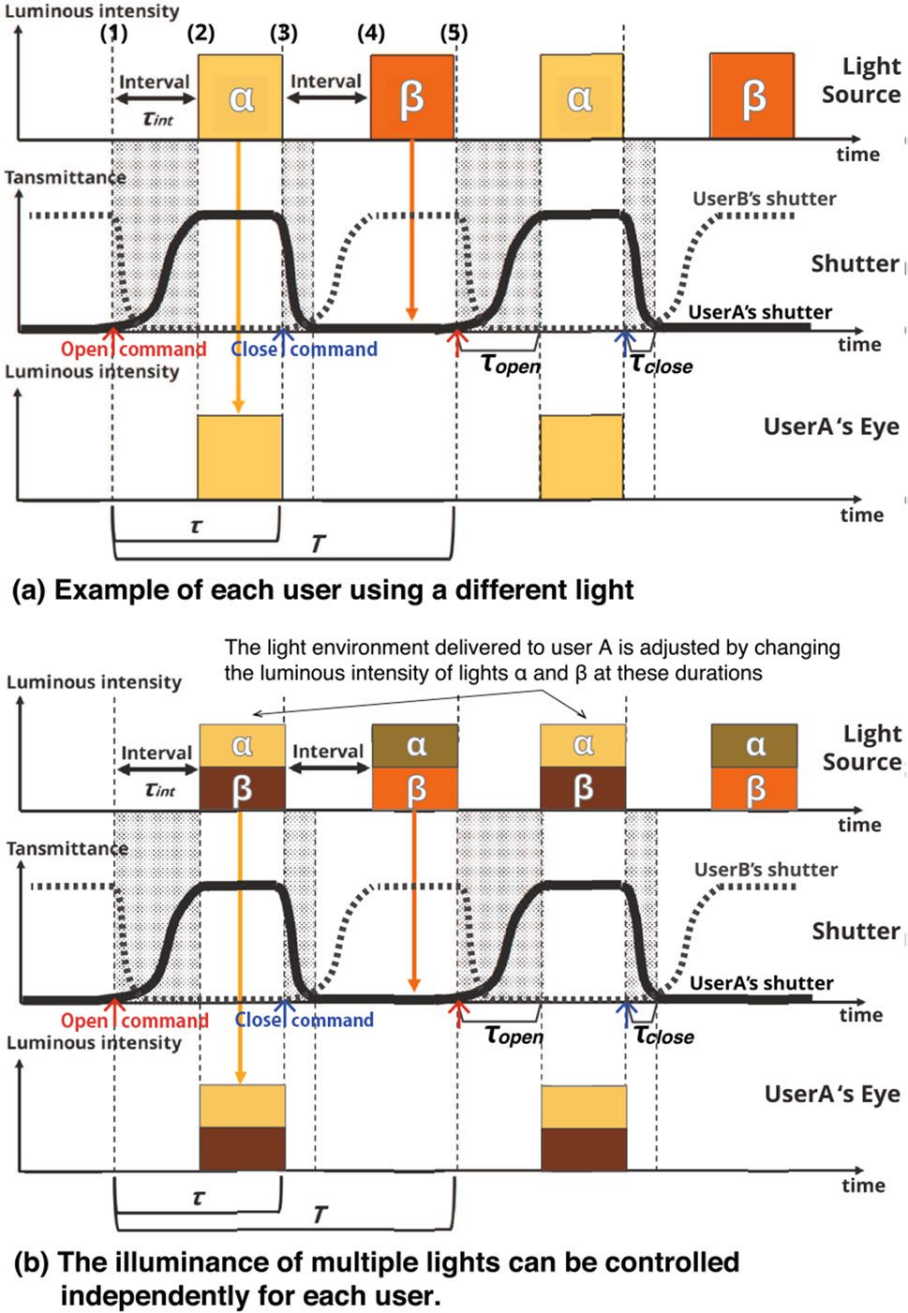
State transition in synchronous control of shutter and light in the proposed system. Example of a system with two users, that is, multiplicity of 2 ($N=2$)

Next, we give a full description of the control algorithm of the proposed system with all the details in Fig. 5, which shows a situation in which the multiplicity is 2, that is, there are two users with independent light environments ($N=2$). For clarity, the time taken to open and close the shutters ($\tau _{\mathrm{open}}$, $\tau _{\mathrm{close}}$) is exaggerated, as shown in Fig. 5.

Figure 5a shows a situation where only light-$\alpha$ reaches User A and only light-$\beta$ reaches User B. In this system, the light source is controlled to blink, and the shutter is controlled to open and close by installing triggers between the LED and constant-current power supply and between the LCD shutter and constant-voltage power supply to turn their connections on and off. The timing of opening and closing the shutter was fixed for each user, and the shutter was controlled such that the timing when it was open did not conflict with other users in one cycle duration *T*.

The following procedure controls User A’s shutter to allow only light-$\alpha$ to pass through. The numbers correspond to Fig. 5a. (1) An open command was sent to the shutter from the shutter driver. (2) After $\tau _{\mathrm{int}}$, light-$\alpha$ is turned on when the shutter is completely open. (3) When a close command is sent from the driver to the shutter, light-$\alpha$ turns off and the shutter starts to close. (4) $\tau _{\mathrm{int}}$ after light-$\alpha$ is turned off, light-$\beta$ is turned on. At this point, the shutter is completely closed and the light of light-$\beta$ does not reach User A. (5) $\tau$ after the close command is sent to the shutter, light-$\beta$ is turned off and the open command is sent to the shutter again.

Regarding User B’s shutter, open and close commands are sent at a time $\tau$ later than that of User A. The time interval $\tau$ between the open command being sent and the close command being sent is determined by the one-cycle duration *T* and the number of multiplicity *N* as follow:

5 $$\begin{aligned} \tau = \frac{T}{N} \end{aligned}$$

Figure 5b demonstrates a situation in which the luminous intensity of multiple lights is controlled independently according to user’s input. In this example, User A wants light-$\alpha$ to be bright and light-$\beta$ to be slightly dark, whereas User B wants light-$\alpha$ to be slightly dark and light-$\beta$ to be bright. The system presents the light environment desired by User A (B) by controlling the light source (the brightnesses of light-$\alpha$ and light-$\beta$ in Fig. 5b) while the shutter of User A (B) is fully open. When the light environment desired by each user changes, the luminous intensity of each light is adjusted for the duration when the lights are on for each user. In this case, each shutter’s opening and closing timing does not change.

## Verification of behavior and indoor environmental assessment in multiplexed lighting environment

To confirm whether the proposed method can improve an indoor lighting environment, we conducted an experiment with participants to clarify the changes in human behavior (H1 in 3.1) and psychological state and evaluated the indoor environment (H2 in 3.1) using our developed system. In this experiment, we constructed two types of environments: a “mono-lighting environment” and a “multiplexed lighting environment.” In a mono-lighting environment (mono-condition), everyone in a shared space experiences the same changes in lighting conditions as those in a normal lighting environment. In a multiplexed lighting environment (multi-condition), the lighting conditions experienced by one occupant change although the lighting conditions experienced by the other occupants do not change; this environment was realized using our system. We then asked the participants to work in both mono- and multi-lighting environments and measured their behaviors, psychological states, and evaluations of the indoor environment. If a decrease in the selected lighting intensity or increase in the number of lighting controls is observed, it means that the occupant potentially has a demand for lighting control that is not sufficiently satisfied in a conventional mono-lighting environment.

The datasets generated and analyzed during the current study are available from the corresponding author upon reasonable request.

### Experiment design

**Fig. 6:**
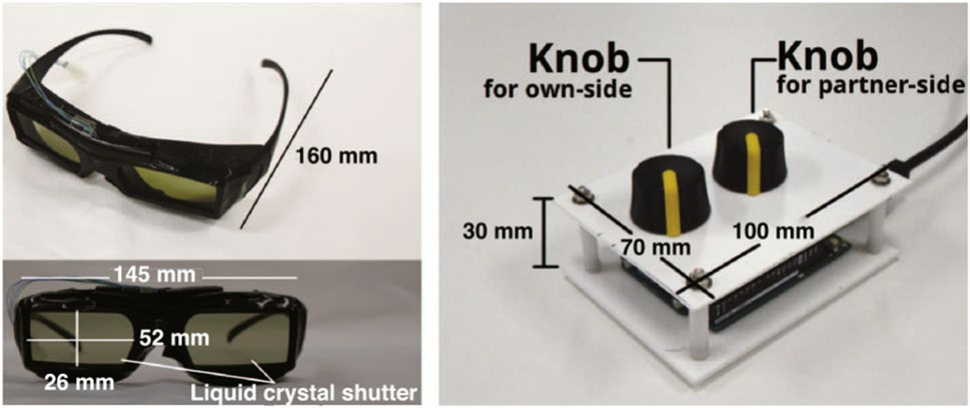
Glasses-type shutter device and lighting controller device

We designed a within-participant experiment, where two participants simultaneously worked on tasks in a single room. Two participants were seated in front of a desk face-to-face and were prohibited from communicating during the experiment. The participants wore glasses-type shutter devices (Fig. 6, left) controlled by our system, and they could manually control the lighting in the room using controller devices (Fig. 6, right) during the experiment. The lighting system was placed with two degrees of freedom on both sides of the participant and the participant’s partner. The participants could control the lighting installed at the top of the desk on each side within a range of 0–310 lx below the lighting. The ordinance on industrial safety and health, which is a ministerial ordinance of the Ministry of Health, Labour, and Welfare that sets forth standards for occupational safety and health in Japan, stipulates standards for the “illuminance” of work surfaces in places where workers are always employed. The regulation states that the standard should be 300 lx or more for “precision work” and 150 lx or more for “ordinary work.” On this basis, we set the illuminance range in this study to satisfy these values. In this case, the illuminance of the opposing side on the desk was 0–77 lx. The illuminance was adjusted to change linearly with the amount of knob rotation.

The participants worked on a typographical checking task called an alphanumeric verification task (AVT) designed to simulate common office work (Boyce et al. 2000). In this study, participants repeated the task of pointing out the difference between two six-character uppercase alphanumeric strings. One character of the two adjacent strings differed, and the participants repeated the findings and markings of these different characters. The characters were printed in 14-point Geneva font. The size and type of the font were the same as those used in a previous study (Boyce et al. 2000), and we confirmed that the participants in this experiment could view the font without any problems. We administered questionnaires before and after the task. Based on the responses to these questionnaires, we evaluated their psychological state and impressions of the indoor environment.

#### Settings of experimental environment

**Fig. 7:**
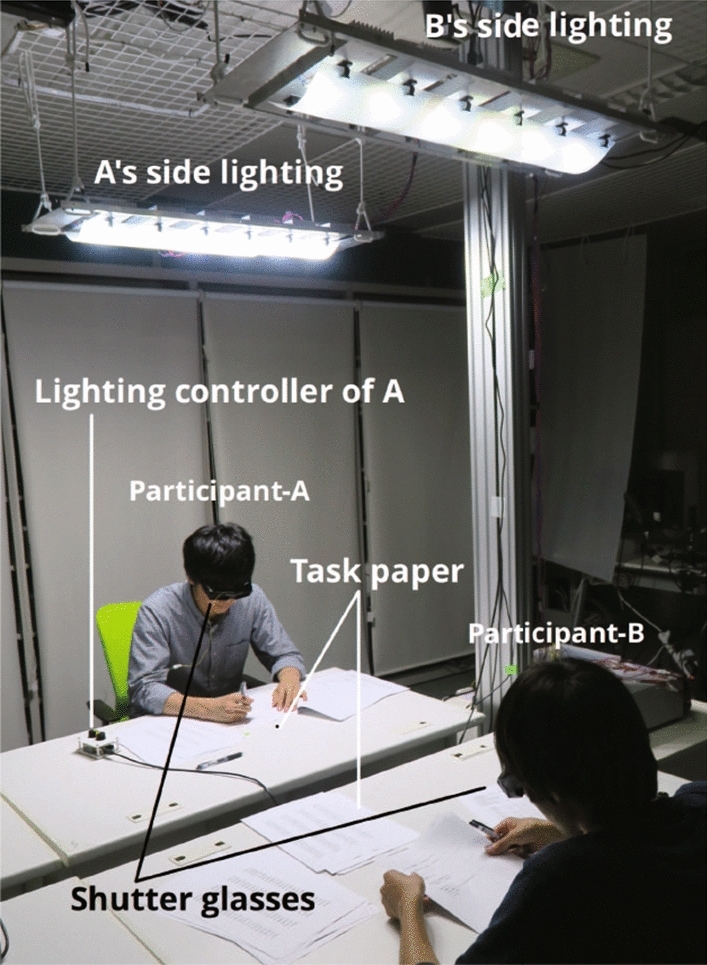
Experiment setting

Figure 7 shows the experiment environment. No light entered the room, and the only light emitted was generated by the illuminator of the system. The experiment was conducted with two desks in a room facing each other, and there were no obstructions to the line of sight between the two desks. The desktop, walls, and screen behind the desk were white. The room temperature was maintained at 26 $^\circ$C.

#### Dependent variables

Each participant attempted the task under the two lighting environments as described below.

- Mono-lighting environment (mono-condition): In this environment, everyone in the space shares changes in the lighting conditions, as in a general lighting environment. When one occupant changes the lighting condition, the lighting conditions change for all other occupants.
- Multiplexed lighting environment (multi-condition): This environment provides the occupants an experience of independent lighting conditions. When one occupant changes the lighting conditions, no changes in lighting conditions occur for any other occupant.

**Fig. 8:**
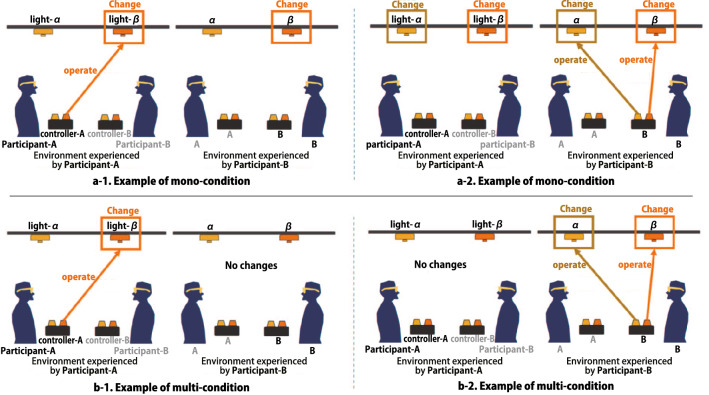
Examples of participants’ experience in mono- and multi-condition

Figure 8 illustrates these two conditions. To describe the situation, we denote the two participants as participants A and B and the light above the work surfaces of participants A and B as light-$\alpha$ and light-$\beta$, respectively. In the multi-condition experiment, the light operation of participant A(B) did not affect the lighting environment experienced by participant B(A) (Fig. 8b-1, 2). In this experiment, each participant experienced each of these two conditions once.

#### Independent variables: behavioral reaction

The control output of the controller device was continuously recorded during the experiment to measure the following index:

- Task performance: Task performance was represented by the number of string pairs in which each participant pointed out a difference. Only the correct answers were counted among the points where differences were pointed out.
- Lighting operation event: For the lighting on both sides, we recorded the number of operation events. The process from starting to turn the knob of the lighting controller device to releasing it was counted as one operation event.
- Selected illumination intensity: For the lighting on both sides, we recorded the illumination intensity while the participants worked on the tasks. The intensity of lighting was recorded on a scale of 0–100, where 0 indicates turning off the lights and 100 indicates the maximum brightness that the prepared lights can provide. When the intensity of illumination was 100, the light alone could provide 310 lx of illuminance on the table surface directly below. For each participant, the average lighting intensity for each side during the 45-min task period was used as the evaluation index.

#### Independent variables: subjective reaction

In this study, all the questionnaires were translated into Japanese because the recruited participants were Japanese speakers. For detailed evaluation items, please see Fig. 12.

- Positive and Negative Affect Schedule (PANAS) in Japanese (Kawahito et al. 2011): This questionnaire provides a means to assess mood based on a model that divides mood into positive and negative. The respondents rated the extent to which they felt each mood, as represented by 20 adjectives on a 6-point scale, where one represents strongly agreeing and six represents strongly disagreeing. The 20 adjectives were divided into 10 items for positive mood and ten items for negative mood, and the sum of the ten values was calculated as the score for each mood.
- Activation-deactivation adjective check list (AD-ACL) (Thayer 1989): This scale is a multidimensional test of transitory arousal states using a four-point self-rating system: “definitely feel” (4), “slightly feel” (3), “cannot decide” (2), or “definitely do not feel” (1). The respondents rated the extent to which they felt each mood, as represented by the 20 adjectives. This questionnaire was scored by averaging five scores for each subscale: “Energetic,” “Tiredness,” “Tension,” and “Calmness.” “Energetic” was determined by the sum of the ratings on the active, vigorous, energetic, lively, and full-of-pep scales. “Tiredness” was determined by the sum of the ratings on the drowsy, sleepy, tired, wide awake, and wakeful scales, the last two being reverse-coded. “Tension” was determined by the sum of the ratings on the tense, jittery, clutched-up, intense, and fearful scales. “Calmness” was determined by the sum of ratings on the placid, at-rest, calm, still, and quiet scales.
- Office environment assessment questionnaire: We used two types of questionnaires to evaluate the subjective assessment of the indoor environment based on previous studies. The first part comprised a questionnaire in which Hedge et al. evaluated office lighting and office environments  (Hedge et al. 1995). The respondents rated the quality of the lighting environment on a 5-point scale based on seven questions and evaluated their satisfaction with the environment, their subjective impression of the lighting, and the work stress on a 7-point scale. The second part comprises three questions regarding the quality of lighting in the room, the ease of adjusting the lighting, and their preference for controlling lighting rated on a scale of one to ten. This questionnaire was used by Boyce et al. (2000)

Finally, we asked the participants to provide an open-ended description of their experiences in the post-questionnaire.

#### Participants

In the paired samples t test, the effect size of each item to be obtained above was estimated to be 1, referring to a previous case, and the sample size was calculated to satisfy the power of 0.8 and a significance level of 0.05 in this case (Dupont and Plummer 1990). As a result, we determined the number of participants to ten. The participants were healthy males with ages ranging from 21 to 24. The ten participants were randomly assigned to five pairs. Six of the participants wore glasses regularly, two occasionally wore glasses, and the final two did not wear glasses according to the pre-questionnaire. The Ethics Committee of the University of Tokyo approved this study (No. 18-179). Written informed consent was obtained from all participants.

#### Operation procedure

**Fig. 9:**
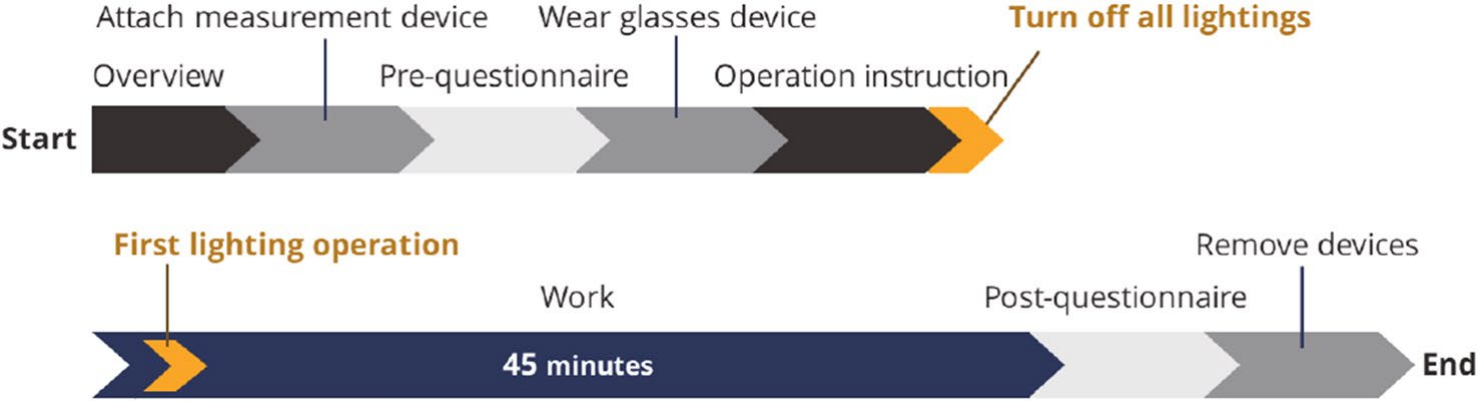
Operation procedure

As shown in Fig. 9, after providing instructions, including an explanation of the lighting device control and the task, we asked the participants to begin working. In the questionnaire given before the task, we asked the participants to respond to the psychological indexes, PANAS and AD-ACL. After wearing the device, the participants were instructed about the operation of the controller and were given time to practice the operation. At this point, the participants were told whether the task environment was mono- or multi-condition; that is, they knew whether their lighting operation would affect the other’s lighting environment. The participants were instructed to control and adjust the lighting conditions so that they felt it was easy to work without restrictions. All lights were turned off prior to starting the task. The participants were then told to control and adjust the lighting after they were given a cue to begin the task. After the end of the task, the participants answered all questionnaires, PANAS, AD-ACL, and other questionnaires for the lighting environment.

All the tasks and questionnaires were printed with black letters on a white sheet of A4 paper and filled with a black ballpoint pen. Boyce et al. (2000) showed that the lighting operation should occur approximately one to three times during a 45-min task operation. Accordingly, the task duration was set to 45 min, and several lighting operation events were expected. The experiment was conducted simultaneously for both participants of each pair. The pairs and procedures were identical for the two lighting conditions, and the trials were conducted on different days. The trial order of the two lighting conditions was counterbalanced among the participants, that is, the pairs of six people and two pairs of four people performed the task in the reverse order of the conditions.

**Fig. 10:**
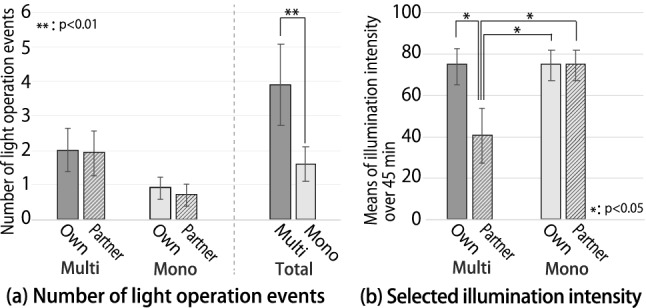
Results of behavioral reaction (means and standard errors, n=10)

### Results

#### Behavioral reaction

- Light operation event: Figure 10a shows the number applied for each side and the total number of operations on both sides. Compared with the mono-condition, the average number of lighting operation events increased more than twice under the multi-condition. The normality of the data was confirmed using the Shapiro–Wilk test, and a paired t test showed that the total number of operations in the multi-condition was significantly higher than that in the mono-condition ($p < .01$).
- Selected the illumination intensity: Figure 10b shows the means of the selected illumination intensity of the light on both sides during 45 min of work in each condition. The relationship between the selected illumination intensity and illuminance is as described in Sect. 4.1.3. Under the multi-condition, the illumination intensity of the light on the partner’s side was adjusted to half that of the other conditions. The results were found to be normally distributed according to the Shapiro–Wilk test at the 5% level. We analyzed the results with a two-way repeated ANOVA test and Tukey multiple comparisons at the 5% significance level. The ANOVA test revealed a marginally significant main effect and medium effect size of the lighting condition (multi and mono) [F(1, 9) = 4.56, $p < .10$, $\eta ^2 = .0336$]; in addition, there was an interaction effect between the lighting condition and the light side (own side and the partner’s side) [F(1, 9) = 6.39, $p < .05$, $\eta ^2 = .0415$]. The post-hoc test revealed that the illuminance of the lighting on the partner’s side under multi-condition was significantly lower than that under the other conditions ($p < .05$).
- Task performance: The mean and standard error of the participants’ task performance in the multi-condition experiment were $1242.8\pm {26.4}$, and in the mono-condition, they were $1286.9\pm {56.6}$. We confirmed the normality of the data using the Shapiro-Wilk test, and a paired t test was performed. This test clarified that there were no significant differences in task performance between the mono- and multi-condition tasks.

**Fig. 11:**
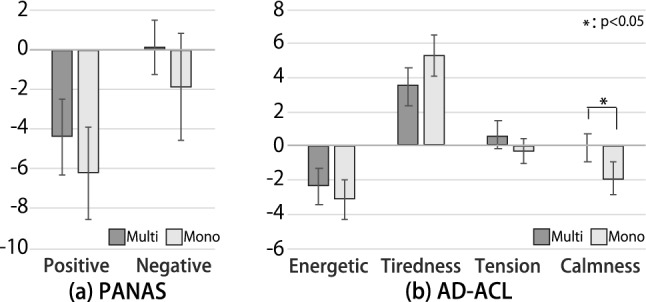
Results of affective evaluation (means and standard errors, n=10). The value is the post-task survey value minus the pre-task survey value

**Fig. 12:**
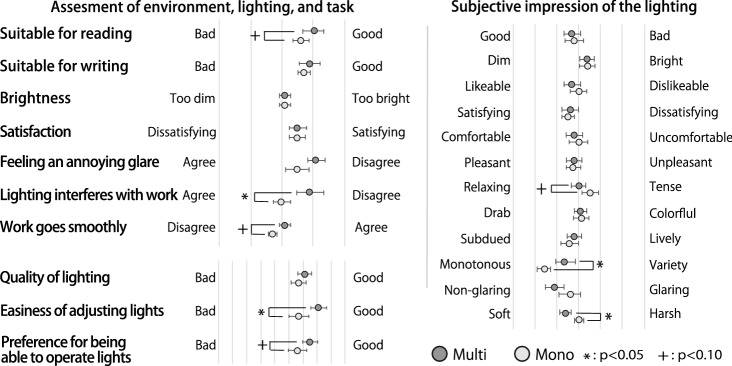
Results of environmental evaluation (means and standard errors, n=10)

#### Subjective reaction

- Psychological evaluation: Figure 11 shows the result of the psychological evaluations. By calculating the difference between the survey results before and after the task, we observed how the psychological behavior of the participants changed with the task under each lighting condition. The PANAS results showed that both the positive and negative scores were lower in the mono-condition than those in the multi-condition. However, the paired t test did not reveal a significant difference in each score between the two conditions. Figure 11b shows the change in each AD-ACL score from pre-task to post-task. Except for “tired,” the score in the mono-condition decreased to a larger value than that under the multi-condition. With a Shapiro–Wilk test, we confirmed the normality of the data, and the paired t test showed that there was a significant difference between the multi- and mono-conditions for the calmness score ($p < .05$), and there were no significant differences between these two conditions regarding the energetic, tiredness, and tension scores.
- Environmental evaluation: Figure 12 shows the subjective evaluations of the indoor environment and the lighting conditions. As these were ordinal scale data, we used the Wilcoxon signed-rank test, which was applied to paired non-parametric data. This test revealed significant differences, as shown in Fig. 12. These tests indicated that the indoor environment in the multi-condition was rated more positively than that in the mono-condition. The participants answered that the multi-condition was marginally significantly more suitable for reading than the mono-condition (from the result of “Suitable for reading” ($p < .10$)), the lighting of the multi-condition did not interfere with the work (from the result of “Lighting interferes with work” ($p < .05$)). The work conducted under the multi-condition went more smoothly than that conducted under the mono-condition (from the result of “Work goes smoothly” ($p < .10$)). Further, for the lighting condition, the simplicity of adjusting the lighting and the preference for operating lights under the multi-condition were rated higher than those under the mono-condition (from the result of “Easiness of adjusting lights” ($p < .05$) and that of “Preference for being able to operate lights” ($p < .10$)). Moreover, regarding the subjective impressions of the lighting itself, the multi-condition had significantly higher ratings in terms of variety ($p < .05$) and significantly lower ratings in terms of harshness than those of mono-conditions ($p < .05$). The test also showed that the participants were marginally significantly more relaxed in the multi-condition than in the mono-condition ($p < .10$).

### Discussion

The experimental results showed that the selected illumination intensity of the partner side was significantly lower in the case of multiplexed lighting environments than that in the case of a mono-lighting environment (Fig. 10b). This result for multiplexed lighting environments is consistent with the findings of a previous study that localized lighting, wherein the ambient brightness is smaller than the brightness of the work surface, increases the degree of concentration (Sakaue et al. 1997). Therefore, it can be concluded that participants potentially demand a lower intensity for partner-side lighting; however, their behavior in controlling lighting was suppressed in a mono-lighting environment. In a shared space, occupants may not be able to express their preferred lighting conditions and they may not be aware of it (Shin and Woo 2009; Niemantsverdriet et al. 2017). Consequently, the introduction of a multiplexed lighting environment can meet the potential demands of the occupants, which may have influenced the setting of the intensity for partner-side lighting.

Furthermore, the number of lighting operation events significantly increased in the multiplexed lighting environments compared with that in the mono-lighting environment (Fig. 10a Total). This result suggests that sharing the lighting environment with others in an indoor environment with multiple occupants, in other words, the interference of the lighting environment with other occupants, inhibits the lighting operation behavior. These results support hypothesis H1; that is, the user’s lighting operation changed. The results of this experiment indicate that realizing a multiplexed lighting environment will meet the demands of the occupants to a greater extent.

The influence of multiplexed lighting environments also extends to the psychological states and subjective environmental assessments of the participants. PANAS results showed that positive values decreased in both the multi- and mono-conditions after the task compared to those before the task, which is natural because the participants were exhausted after the 45-min task. On the other hand, the negative values decreased from the pre-task to the post-task, that is, in the positive direction, suggesting that the task was not sufficiently unpleasant to cause severe discomfort. Moreover, it is difficult to discuss the trend in the PANAS results because both the positive and negative scores in the mono-condition were slightly lower than those in the multi-condition (Fig. 11a). In contrast, the AD-ACL results showed that the calmness score was significantly higher under the multi-condition than under the mono-condition (Fig. 11b). In addition, several environmental assessments were rated significantly higher under the multi-condition than those under the mono-condition.

Participants felt that the multi-condition was significantly less disruptive and easier to adjust the lighting than the mono-condition. Although marginally significant, participants found it easier to read and work more smoothly in the multi-condition than in the mono-condition (Fig. 12). The subjective evaluations showed that the lighting impressions were more relaxed, diverse, and softer under the multi-condition than under the mono-condition. The differences in the subjective impressions of these two conditions were related to the participants’ behaviors, such as the number of lighting operations and the set illuminance. In the multi-condition, the participants lowered the illumination intensity of their partner’s side, which may have softened their impression of the lighting and made the environment easier to work in. In addition, the fact that the number of lighting adjustments during the task was higher in the multi-condition than in the mono-condition seems to be related to the response to “Easiness of adjusting lights.”

Previous studies have shown that the ability of an individual to control lighting in a room enhances the evaluation of the indoor environment (Juslén et al. 2005; Moore et al. 2004; Afshari and Mishra 2015). Evaluation of the multi-condition, which allows an individual to control lighting independently of others, was improved compared to the mono-condition, which is consistent with previous findings. These results support hypothesis H2 whereby multiplexed lighting can improve occupants’ reputation in an indoor environment and their psychological conditions.

In summary, our results suggest that the multiplexed lighting system can eliminate the suppression of the desired lighting operation caused by sharing a lighting environment with others and allows individuals in a single space to easily set their own desired lighting environment. This change in lighting operation improves the impression of the indoor environment and occupants’ psychological conditions. Conventional intelligent lighting methods have only a limited number of ways to deal with the conflicting lighting needs of two nearby occupants; balancing the two to improve overall space satisfaction (Bando et al. 2018), prioritizing the lighting needs of the person who was there first (Petrushevski 2012), and so on. As an approach other than lighting adjustment by the system, it has been proposed that when a person changes the lighting environment, the user should be given confident that the change is acceptable to others, thereby reducing the sense of avoidance of lighting adjustment (Niemantsverdriet et al. 2017). In contrast, the multiplexing approach avoids the conflict of desire for lighting itself in our research.

One of the limitations of multiplexing lighting is the number of people who can simultaneously experience the system. Previous intelligent lighting methods have achieved system operation in environments with more than a dozen people in the same space (Kar et al. 2019). However, our approach is highly effective when two or three people are present in a small room. For example, it has been reported that lighting conflicts can occur even between couples living in studio apartments  (Niemantsverdriet et al. 2017). In particular, remote working from home has spread rapidly in recent years, partly because of the worldwide spread of the coronavirus disease (COVID-19) pandemic since the beginning of 2020 (Gallacher and Hossain 2020). For this reason, conflicts of lighting desires are expected to increase; for example, one person may desire a break from work, while the other wants to concentrate on work. The proposed method is likely to play a critical role in such situations.

## Limitations and future works

As described above, the constraint of multiplicity, which is the number of lighting environments that can be simultaneously achieved, is one of the limitations of this study. As discussed in Sect. 3.3, the maximum value of the multiplicity depends on the opening and closing speeds of the shutter. In the present system, using an LC shutter, a maximum multiplicity of three could be achieved. Because of the limitation of the current shutter opening/closing speed, if the multiplicity is increased, the duration in which the light reaches the eyes of each person will be too short, resulting in a darker working environment. To meet the standard of illuminance of the work surface, the luminance of the original lighting must be extremely high. To address multiple constraints, we should introduce a shutter with faster opening and closing times. Ferroelectric liquid crystal and MEMS shutters may be used in the future as shutter elements that can open and close faster than the LC shutters used in this study.

In addition, we did not confirm the effects of light multiplexing on task performance in this study, although changes in the lighting behavior were observed. However, the subjective impressions of lighting and indoor environment improved, and thus, the task performance can be expected to improve over long hours if users conduct tasks in a multiplexed lighting environment.

Furthermore, in this study, although light multiplexing changes only the brightness, we believe that the colors of the lights can be multiplexed using our proposed approach. Multiplexing lighting colors can realize a multiplexed lighting environment, where occupants who want to relax are presented with low-temperature lighting and occupants who want to concentrate on their work are presented with high-temperature lighting. We examined the effectiveness of the proposed approach in a situation in which several workers cooperated in an office. In the future, it is expected that the proposed approach can be applied to situations in which various user states, such as relaxation and concentration, coexist.

In this study, we investigated the influence of our system in the context of office work; however, applying our system to a living environment at home or at rest in an office environment is conceivable. Moreover, the multiplexed lighting environment can be applied to a wide range of lighting applications, for example, entertainment, such as stage lighting, with different lighting conditions of the same scene for different audience members, or a light guidance display providing different guidance to different viewers.

## Conclusion

An indoor lighting environment affects different occupants individually, and a method to satisfy all the lighting demands of different occupants is yet to be realized. In this study, we proposed and developed a lighting system that realizes multiple lighting environments in a single space to simultaneously meet the different lighting environment demands of different occupants. The developed system applies time-division light multiplexing by controlling the shutter and the light source. It realizes multiple illumination states that are independent of each other in a single space. We conducted an experiment to clarify the effects of our system on the occupants within an environment.

In the multiplexed lighting environment, where occupants can control lighting without affecting other occupants, the number of light operations increased and the selected illumination intensity of the area around the work-space decreased. These results suggest that sharing a lighting environment with other occupants suppresses the potentially required behavior of lighting control. Our proposed system enables occupants to be free to satisfy their individual requirements for different lighting environments against such suppression. In addition, the impressions of the lighting environment were positively affected by multiplexing the lighting environment.

The concept of multiplexed lighting can bring new possibilities to the field of illumination. As described in the Limitations and Future works, further development of this concept can be expected as hardware advances and applications expand.

## Data Availability

As we are continuing with the research, data is not submitted, but it can be given at a genuine request.
